# Developing and Implementing Multidisciplinary Service Packages and Pathways in the Finnish Wellbeing Services Counties

**DOI:** 10.5334/ijic.9541

**Published:** 2026-11-04

**Authors:** Juha Koivisto, Eeva Liukko, Heidi Muurinen

**Affiliations:** 1 Finnish Institute for Health and Welfare, Finland

**Keywords:** integrated care implementation, multidisciplinary service packages and pathways, governance and management, health and social care reform

## Abstract

**Introduction:**

Integrated care for clients with complex needs requires structured approaches that support coordination across sectors, professions, and organisations. In Finland, the 2023 health and social care reform established wellbeing services counties and introduced a statutory requirement to organise services as multidisciplinary service packages and pathways.

**Case description:**

This article examines the development and early implementation of multidisciplinary service packages and pathways in Finnish wellbeing services counties. A co-creation model for defining, steering, and monitoring service packages and pathways is used as an analytical framework. The study combines a national-level questionnaire (16 counties) with in-depth group interviews conducted in three counties focusing on services for neurodivergent children.

**Findings:**

Development work was uneven and at early stages. Defining service packages and pathways helped clarify client groups, shared goals, and collaboration practices, but steering and monitoring systems remained weak. Key implementation challenges related to governance, professional cultures, data availability, and coordination across sectors.

**Lessons learned:**

Multidisciplinary service packages and pathways can support the planning and delivery of individualised service pathways, but only when embedded in management structures, supported by leadership, and linked to monitoring and learning mechanisms.

**Transferability:**

While shaped by Finland’s reform context, the findings provide transferable insights into implementing integrated care through predefined service structures in other health and social care systems.

## Introduction

Integrated care aims to improve the coordination, quality, continuity, and effectiveness of services for individuals with complex and multidimensional needs by overcoming fragmentation across professional, organisational, and sectoral boundaries [1,2]. Despite widespread policy commitments to integration, many health and social care systems continue to struggle with siloed service structures, unclear responsibilities, and weak mechanisms for coordinating care across sectors — particularly for clients requiring support from multiple services simultaneously, who are at greatest risk of fragmented pathways, repeated assessments, and gaps in continuity of care [3,4]. A relatively small proportion of service users account for a disproportionate share of service use and costs, and integrated care has therefore been promoted as a key strategy to improve both client outcomes and system sustainability [5]. However, translating integration principles into concrete practice remains challenging, particularly in systems characterised by strong professional boundaries and institutional separation between health and social services.

In Finland, these challenges have been well documented. Previous studies show that approximately 10% of service users account for a substantial proportion of health and social care costs, while often receiving poorly coordinated services [6,7,8,9]. Although the Finnish health and social care system has traditionally been considered relatively integrated by international standards, practices for service integration have varied considerably across municipalities and service sectors [10,11]. Prior to 2023, responsibility for organising services was divided among nearly 300 municipalities and twenty-one hospital districts, resulting in fragmented governance, uneven access to services, and limited capacity to coordinate multidisciplinary care, particularly in smaller municipalities [12].

To address these challenges, Finland implemented a major health and social care reform in 2023. Responsibility for organising public health and social services was transferred from municipalities to twenty-one newly established wellbeing services counties, with the aim of strengthening equity, continuity, and service integration at the regional level [13,14]. Only Helsinki retained its existing service structure; in this paper, “county” also refers to Helsinki. The reform created structurally integrated organising bodies with responsibility for both health and social services and introduced direct state funding to support regional coordination. Importantly, the legislation explicitly obliges the counties to organise services as integrated service packages and pathways for clients who require multidisciplinary support.

While the reform established favourable structural conditions for integration, it also created an immediate need for practical tools and models to support the design, coordination, and governance of integrated care in everyday practice. Structural integration alone does not guarantee functional integration at the level of service delivery, professional collaboration, or client experience [5,15]. Recent studies conducted shortly after the reform indicate that the maturity of service integration remains uneven across counties and that concrete governance and coordination practices are still developing [16,15].

In this context, multidisciplinary service packages and pathways have emerged as a key approach to operationalising integrated care in Finland. Service packages refer to predefined sets of services designed for specific client groups and delivered by multiple providers, often simultaneously. Service pathways refer to preplanned sequences or combinations of services that structure how care and support are provided over time. Together, service packages and pathways are intended to support the planning and delivery of individualised service pathways by clarifying included services, responsible actors, and modes of collaboration in advance [17,18].

This article presents an empirical case of how multidisciplinary service packages and pathways have been developed and implemented during the early phase of Finland’s health and social care reform. The focus is on implementation processes rather than policy design: how integration is translated from legislative and strategic objectives into concrete service practices within newly established governance structures. The study draws on a two-phase research design combining a national-level overview of development work across wellbeing services counties with in-depth case analysis of service packages and pathways developed for neurodivergent children.

As the analytical framework, the study applies the THL co-creation model for defining, steering, and monitoring service packages and pathways [17]), which conceptualises service integration as a continuous, iterative process emphasising shared understanding, governance, and knowledge-based management. This article aims to contribute to the integrated care literature by providing empirical insight into how integrated care practices develop in the context of a major system-level reform, illustrating how predefined service packages and pathways function as implementation tools, and identifying governance challenges and enabling factors relevant beyond the Finnish context.

## Description of the care practice

### Context and rationale

The 2023 Finnish health and social care reform transferred responsibility for organising public health and social services from municipalities to twenty-one wellbeing services counties, each accountable for coordinating services across health care, social welfare, and, through collaboration, municipal and third-sector services [13]. Only Helsinki retained its existing structure. A central objective was to strengthen integration for clients with complex, multidisciplinary needs. However, integration at service delivery level had historically been uneven, with fragmentation a persistent challenge — reflected in overlapping assessments, parallel care plans, unclear responsibilities, and discontinuities in care [10,11,19,20].

The reform created new structural conditions by consolidating organisational responsibility at the regional level, but early research confirms that structural integration does not automatically improve coordination or continuity of care in practice [15]. The counties have therefore faced an urgent need for concrete tools to support integrated care across services, professions, and sectors.

Within this context, multidisciplinary service packages and pathways were introduced as a key care practice to operationalise integrated care. They are intended to provide a structured means of organising and coordinating services for defined client groups while supporting the planning of individualised service pathways. The care practice responds both to legislative requirements and to long-standing practical challenges in service integration identified in earlier Finnish and international research.

### The care practice: service packages and pathways

Under the Act on the Organisation of Health and Social Services [13], counties are obliged to deliver health and social services as integrated packages and pathways and to integrate these with municipal, state, and third-sector services. A service package comprises a set of services organised for a specific client group and often used simultaneously (e.g. employment, social work, and rehabilitation services). A service pathway is a pre-planned sequence of services provided consecutively or partially concurrently, so that one service precedes another in a structured order. Together, they form the basis from which individualised service pathways are assembled for each client [17,18].

Service packages and pathways are not intended to replace individualised care planning but to function as a framework within which personalised service pathways can be designed and delivered. The underlying assumption is that individualised service pathways can be more effectively realised when relevant services, responsible actors, and modes of collaboration are defined in advance for client groups with similar patterns of need. This approach aligns with international integrated care literature emphasising the importance of care pathways, coordination mechanisms, and shared frameworks in supporting person-centred care for people with complex needs [4].

In practice, the approach involves: identifying client groups with complex or multidimensional needs; defining shared goals from client, professional, and system perspectives; clarifying roles, responsibilities, and cross-organisational collaboration practices; and supporting individualised service planning by giving professionals a coherent overview of available services and agreed coordination arrangements.

## Co-creation model as a framework for analysis

Between 2018 and 2020, THL and the future counties developed a co-creation model for defining, steering, and monitoring service packages and pathways [17], which serves as the analytical framework for this study. The model rests on the premise that effective individualised service pathways require pre-defined packages and pathways as a foundation, assembled through collaboration among service organisers, providers, professionals, and clients.

The model comprises three interactive and iterative stages. In the definition stage, actors analyse the local service system and client needs, identify target client segments, agree on shared goals and indicators, specify the required services, and establish rules for cross-organisational cooperation. In the steering stage, actors agree on how to direct service delivery towards the defined goals, selecting appropriate steering methods (e.g. information-based or resource-based) and establishing dialogue mechanisms to build mutual trust and support self-management. In the monitoring stage, actors agree on how to track whether services are organised and delivered according to plan, using quantitative and qualitative indicators defined in the first stage and establishing responsibility for analysing and acting on monitoring data.

## Study design and data

The study draws on a two-phase empirical design conducted in 2023, combining a national-level overview of early implementation across wellbeing services counties with in-depth case analysis of service packages and pathways developed for neurodivergent children. The co-creation model [17] served as the analytical framework. The focus was on understanding implementation processes rather than evaluating outcomes, as most packages and pathways were still at early stages of development.

### First phase of the study: the state of development of service packages and pathways

The study’s first phase (spring 2023) [21] examined the state of development and implementation of the service packages and pathways in the counties: how their definition, steering and monitoring had been planned or were being carried out (Figure 1). The counties were sent a questionnaire that had been pre-tested in one county. Responding to the questionnaire was voluntary for the counties, and no sensitive information was asked.

**Figure 1:**
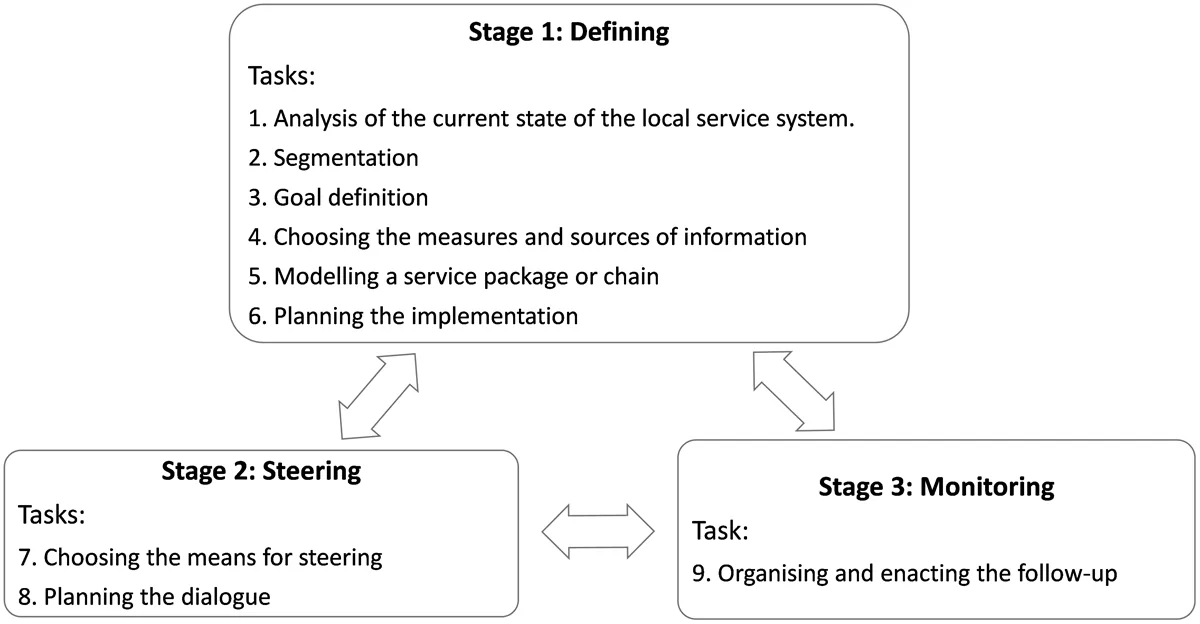
The model for defining, steering, and monitoring service packages and pathways.

The counties were asked to choose 2–5 local service packages or pathways, and to respond to questions regarding the development work. The questions covered the starting points and needs for developing multidisciplinary service packages and pathways, the target groups, the goals, and the current state of implementation. The counties were also asked which service groups were included in the service packages or pathways and what challenges or problems, insights, and successes they had encountered while developing and/or implementing them.

Responses were received from sixteen counties, with 33 respondents. The respondents were predominantly drawn from the professional management or administration of health and social services. They also included project, planning, research, and development professionals. Two respondents were employed in quality management.

The results of the survey are not comprehensive as five counties did not respond to the survey. Also, the survey asked each respondent to provide information on up to five service packages or pathways, so it does not cover all development work. The number of respondents per county ranged from one to four, so some counties provided more descriptions of service packages and pathways than others. As the main objective was to obtain information on the overall state of service integration, rather than by county, we did not consider this to be relevant to the study.

The questionnaire was structured around the co-creation model’s three stages. As the counties had only been established at the start of 2023, development work was at an early phase, so steering and monitoring were not directly asked about; respondents could however address these in open-ended responses. The survey thus provided varying degrees of information across the model’s stages.

The respondents reported 67 multidisciplinary service packages or pathways. Of them, 18 were being defined but had not yet started to be tested or piloted, 14 were in a trial or pilot phase, 21 were in the implementation process, and 14 had been adopted as part of day-to-day service activities with clients. In the following, we describe the responses to the questionnaire by the three stages of the model.

### Second phase of the study: developing service packages and pathways for children with neurodivergent conditions

In the study’s second phase (autumn 2023) [22], the service packages and pathways for neurodivergent children in the wellbeing services counties of Pirkanmaa and Päijät-Häme, and Helsinki were selected for further analysis of a case study [23]. This client group was selected based on the responses to the questionnaire and because of the multisectoral and multidisciplinary nature of neurodiversity services. There is also an evident need and interest in Finland to develop neurodiversity services for children and young people [24,25,26]. The aim was to find out how the service packages and pathways were being developed and whether there were elements in the packages or pathways that could be scaled up between counties or nationally.

Thematic group interviews were conducted in each region with 6–14 participants, for a total of 31. The counties granted research permits to conduct the interviews. In addition, the voluntariness of each interviewee was verified by asking for informed consent from the participants before the interview. The interviews lasted between 86 and 131 minutes in total. They were recorded and transcribed (106 pages, Times New Roman 11 font, 1.5 line spacing). The interview quotes have been translated into English. The language has been slightly clarified in places to make the quotes more readable without altering the meaning of the quotation. To protect the privacy of the interviewees, the counties are referred in random order as County 1, County 2, and County 3.

The participants had been involved in developing or implementing the service packages or pathways. The participants included a range of healthcare, student welfare and social welfare professionals, managers and developers, and people with lived experience. In the interviews, people with lived experience provided information about what kind of support clients and their families need, what difficulties they face in the service system, and how these problems should be solved. They were involved in the local development processes of service packages and pathways, so they had up-to-date information about the regional challenges and the solutions being sought.

The interview guide was in line with the model for defining, steering, and monitoring service packages and pathways. We asked the interviewees how, why and with whom the service packages or pathways were developed, designed, implemented, and managed. We also asked about professional cooperation and how the coordination of services had worked for the clients. Finally, we asked the interviewees for their opinion on to what extent the service packages and pathways were transferable as such to other counties. The first phase of the study (questionnaire responses) influenced the interviews in the study’s second phase. The responses enabled us to steer the discussion towards the issues that were found to be essential and to the possible challenges and strengths of multidisciplinary development work.

Documents describing the development work were requested from the three counties as background material. Only a few documents were available, which may reflect the early stages of development. These documents included descriptions and slideshow presentations of the development work and the agreed-upon models of action.

We used deductive content analysis to analyse the interviews [27,28]. We started with a data-driven approach, with each of the authors classifying one third of the data. The co-creation model served as a framework for the analysis, and it formed a categorisation matrix with main and sub-categories for further analysis, like the one used to analyse the survey. The data was then organised according to the matrix.

Given the early stage of implementation, the analysis did not aim to assess outcomes or effectiveness of service packages and pathways. Instead, it provides an exploratory and descriptive account of implementation processes, challenges, and emerging practices. This approach is consistent with the study’s aim to contribute to understanding integrated care development in context and to identify lessons that may inform future implementation and research.

## Results

### The state of development of service packages and pathways

#### Analysis of local needs and starting points

The respondents identified several needs and starting points for the development of service integration, which can be divided into those related to the service system (organising services) and those related to the client (delivering services). The main challenges for organising services included fragmentation of services and problems in perceiving them as a whole, ambiguities in sectoral and organisational responsibilities and divisions of labour, difficulties in accessing services, and rising demand for and cost of services. There was also a need to strengthen the skills of professionals, improve early risk identification and prevention, and promote multidisciplinary cooperation. Counties also wanted to harmonise work practices and regional approaches. The main challenges and needs in service delivery included repeated assessment of service needs in different services, multiple service and care plans for clients, queues and lack of services, challenges in the timeliness of services and the need to understand the client’s situation holistically.

#### Segmentation

More specific groups were also identified, including adults at risk of or living with diabetes, clients with functional impairments, those requiring emergency assessment or hospitalisation, people in palliative care, those with intellectual disabilities, and clients with financial difficulties or histories of abuse.

#### Goal definition

Goals emphasised early intervention, a shift from intensive to preventive services, enhanced quality and effectiveness, multidisciplinary cooperation, cost containment, and harmonised work practices. From the client perspective, key goals included early identification and referral, joint service and care plans, equal and timely access, client inclusion, and low-threshold services.

#### Modelling the service packages and pathways

To model the service packages and pathways, the services must be identified with the necessary level of detail, as well as the providers and professionals who deliver them. Most commonly, according to respondents, service packages and pathways consisted of mental health services, substance abuse and addiction services, primary healthcare outpatient services, and social services for working-aged people. Other service sectors that were frequently used were specialised psychiatric services and somatic care, health promotion, medical rehabilitation, social services for families with children, disability services, and child protection services. Respondents also acknowledged the importance of NGOs and religious associations, including client organisations and the social work of the church. The services utilised depended on the client group for which the service package or pathway was being designed.

Respondents were asked if the rules and principles for coordinating the included services had been defined. They had been defined for 41 of the 67 service packages or pathways and were already in place for 20 service packages or pathways. In five service packages or pathways this task had not yet been started. One county did not provide an answer.

#### Choosing the sources of information and planning the implementation

Information on steering and monitoring was sparse in the survey responses, as many counties were still at planning or pilot stage and specifying information sources or full-scale implementation was not yet timely. Respondents described steering and monitoring in general terms or not at all, though they did identify challenges encountered in developing and managing service packages and pathways.

#### Challenges and problems

Contrary to the goals of the reform, the transition from municipalities to wellbeing services counties had slowed down the development of service integration and multidisciplinary service packages and pathways, even though this development had already begun in the early stages of preparations for the transition. Responses indicated that finding time to develop service integration was difficult and that there were no existing county structures in place into which service packages and pathways could be prepared and transferred.

Managing a multidisciplinary network of experts was seen as a challenge, and it was not easy to engage employees around a common goal. Service packages and pathways were sometimes so large that they required joint discussions and agreements between managers at different levels and in different sectors and organisations on issues such as shared goals, funding, resources, and approaches. It was stated that actors may have their own agendas and aligning them around common goals was sometimes considered time-consuming and challenging.

An organisation-centred work culture and strong professions challenge the development of multidisciplinary service packages and pathways. The work of other sectors was not always recognised, and there was a need to move from silo working to multidisciplinary working. Interventions that cross sectoral or organisational boundaries (e.g. resource shifts, referral guidelines) were often perceived as being imposed from elsewhere. It was understood that guidelines alone are not enough to change practical work and do not automatically provide a better service to the client.

A change in the management paradigm was recognised as necessary to enable service packages and pathways to function as tools for the service organisers. The need for knowledge-based management and governance was also identified. Management involvement, support, feedback, and encouragement were all considered vital to developing service packages and pathways and a prerequisite for success. Respondents found it useful or necessary to assign process ownership to someone in management with adequate decision-making authority to manage multidisciplinary service packages and pathways.

Respondents noted that cost and effectiveness data on service integration was difficult to obtain and that measuring outcomes was generally challenging. The exchange of information was also hampered by a variety of different information systems. Currently, client data cannot be transferred between sectors. The development of interoperable information systems and the ability to share client information were identified as being essential for integrated service packages and pathways.

Despite these challenges, the importance of working together was recognised, and the development work had laid foundations for more client-centred services with increased client involvement.

### Developing service packages and pathways for children with neurodivergent conditions

#### Identifying local needs and starting points for development work

Neurodivergent children’s services are a large entity, and it can be challenging to determine which activities and services should be included. However, the interviews showed a clear understanding of the starting points for the development work and the tasks to be carried out. Services for children with neurodiverse conditions were found to be fragmented, resulting in fragmented neuropsychiatric service pathways and insufficient support for parents. Children could be left without the long-term rehabilitation they needed, and transfers between services were not always successful. Waiting times for services were also reported. Prior to the health and social care reform, local authorities in the municipalities had implemented different policies on where a child’s individual service pathway began and where neurodivergence was diagnosed. This diversity between municipalities was evident in the newly formed counties, which aimed to harmonise these practices. It had also become clear that parents had not been sufficiently consulted about their child’s issues and had not received sufficient support.

Clients were sometimes referred unnecessarily to specialist healthcare when appropriate help could have been found in primary healthcare and basic social welfare services. There were shortcomings in the competence, cooperation, and working methods of professionals. Separate client information systems in healthcare and social welfare and difficulties in exchanging information between professionals made collaboration difficult, and, for example, previous diagnostic data may not have been available. The need to strengthen the service pathway from the perspective of early support and primary and basic services was obvious.

#### Goal definition

The goals of the development work were formulated similarly in two of the three counties. In County 1, the aim was to strengthen the early support and rehabilitation of neurodivergent children and their parents, strengthen the skills of professionals, and clarify the overall service system and responsibilities of the different actors along the service pathway. In County 2, the aim was to create a child- and family-centred service pathway, with services clustered around the family as needed. The aim was also to provide adequate early support for children and families and to extend the service pathway from early childhood education to specialised healthcare so that services could be provided as needed. The importance of basic services alongside specialised services was stressed.

A further aim was to avoid over- or under-diagnosis and medicalisation. Cooperation and regional coherence were also identified as goals.

The goals of County 3 were somewhat more precise as they focused on the service pathway for children with a suspected ADHD diagnosis. The objective was to determine 1) the distribution of responsibilities for diagnosing suspected ADHD in school-age children between school health care, primary child and adolescent mental health and substance abuse services, and primary pediatric specialist services; 2) how ADHD diagnosis might be carried out more comprehensively in school health care; 3) agree on consultation and other adequate support between specialised healthcare and school health care; and 4) harmonise practices on what should be done before and after the diagnosis process.

#### Modelling the service packages and pathways

The development work was linked to the wider development of mental health or family centre services, which provided a supportive organisational context. The development work in the counties followed a similar, multidisciplinary, multi-professional, and cross-sectoral approach. The development teams comprised management and professionals from primary health care, social welfare, specialised healthcare, educational services, and various development projects. People with lived experience were also involved. Staff were involved, for example, in workshops.

The preliminary descriptions and definitions of the service pathways followed this comprehensive approach. They included professionals from different sectors of the child’s everyday life, such as child health clinics, daycare, schools, primary healthcare and social welfare services, child protection services, specialised healthcare services, and NGOs. The rehabilitation services ranged from everyday activities to demanding medical rehabilitation. However, the aim was to strengthen the support for children and their families in their daily lives, so that specialised healthcare would provide flexible and timely support and consultation to basic services. Parental involvement was considered necessary, and support for parents was seen as an integral part of the service packages and pathways that should be available from public services and NGOs.

#### Transferability of service packages and pathways

One of the study’s aims was to determine whether and to what extent a service package or pathway developed in one county could be scaled up or replicated in another county. Based on this study, the challenges underlying the development of the service packages and pathways for neurodivergent children were similar in all three counties. As a result, the essential aspects, solutions, and modelling details were similar. It can be assumed that these service packages and pathways are likely to contain, or should contain, some common elements for the services to be effective, regardless of location or context. The interviewees considered national models possible.

Defining these necessary elements at a national level may help ensure equal services for neurodivergent children across counties. It may also save time and resources if each county does not have to start from scratch. National guidance was also seen as useful for implementing effective practices.

Based on this study, some possible key characteristics and elements of neurodivergent children’s service packages and pathways could be identified as follows: 1) diagnosing (who makes the diagnosis, where and when); 2) appointing a dedicated worker who is responsible for care and support for the child and family; 3) coordinating needs assessments, and service, care and rehabilitation plans; 4) involving and consulting the family in the child’s affairs, 5) providing the child and family with appropriate support and rehabilitation services in their everyday life, and 6) providing basic and primary services with timely support from specialist healthcare and other specialised services.

#### Steering and monitoring

The service packages and pathways were still being modelled and were not yet piloted or implemented. Developing unambiguous solutions and services was challenging due to differences in organisational cultures, coordination of information systems, information sharing, and variations in management, steering, and monitoring structures. Therefore, choosing the measures and sources of information to steer and monitor service integration was not timely.

Still, the interviewees reflected on the challenges of managing service integration. They emphasised the need for multidisciplinary network management, where the responsibilities of the different organisations are identified, and co-management practices are agreed on. The importance of joint forums and cooperation between municipalities and counties was stressed.

The fact that management and budgets are spread across sectors and organisations was mentioned as a particular challenge, and the need for horizontal support from managers to promote the development and deployment of service integration was considered necessary. To overcome these difficulties, the need to appoint a process owner for the service package or pathway with sufficient decision-making authority was considered essential. This need was also highlighted in the survey responses.

## Discussion

This study examined the development and early implementation of multidisciplinary service packages and pathways in Finnish wellbeing services counties during the initial phase of a major health and social care reform. The findings highlight both the potential of structured approaches to support integrated care and the practical challenges of translating legislative and strategic objectives into everyday service practices. In line with international research on integrated care, the results suggest that integration is not a linear or technical process but a complex, context-dependent endeavour shaped by governance structures, professional cultures, and local implementation capacity [29,5].

Most service packages and pathways were still at early stages of definition or pilot implementation, reflecting both the short time since the counties were established and the simultaneous demands of restructuring governance, budgets, and service provision. Similar patterns are observed in other large-scale reforms, where early phases focus on organisational consolidation while functional integration develops more gradually [30,31].

### Positioning the case in international context

Internationally, integrated care reforms have followed diverse trajectories, ranging from comprehensive structural integration to softer coordination mechanisms and network-based collaboration. Experiences from across Europe and beyond illustrate both the potential and the persistent difficulties of implementing predefined service structures for clients with complex needs.

In the United Kingdom, integrated care pathways were introduced into the NHS from the 1990s onwards and have since become one of the most widely used mechanisms for formalising multidisciplinary collaboration. Systematic reviews confirm that care pathways can be effective in supporting proactive care management for clients with relatively predictable care trajectories, and may bring about improvements in service quality and professional behaviour, though evidence remains mixed, particularly for clients with more variable and complex needs [32]. A key lesson from the UK experience is that top-down strategies that do not actively involve the relevant professionals have little or no impact; engagement of both clinical and management staff in the development and adoption of care pathways is required, and a setting-specific implementation strategy is most likely to be effective [33]. Northern Ireland has a long history of structurally integrated health and social care but continues to face challenges related to professional silos and service coordination at the local level [30], while Scotland’s model of Integration Joint Boards emphasises joint governance and strategic commissioning, while allowing considerable local variation in implementation [34].

In the Netherlands, the government introduced bundled payments for chronic disease management from 2010, incentivising integration through predefined care standards — structured service packages for specific patient groups such as those with diabetes, COPD, or cardiovascular conditions. These care standards describe the desired organisation and quality requirements of functions and services, and include indicators for measuring, monitoring and improving quality of care [35]. However, this single-disease approach proved less fit for purpose for patients with multimorbidity or complex social needs, prompting ongoing initiatives to broaden the scope towards truly person-centred integrated care [36]. Persistent implementation challenges in the Netherlands have included reducing system complexity for citizens and professionals, coordinating between disciplines and organisations, and implementing integrated client records for information sharing [35].

Evidence from the pan-European SELFIE project, which studied 17 integrated care programmes for people with multi-morbidity across eight countries, provides directly relevant cross-national insights. Successful implementers commonly adopted an incremental growth model rather than a disruptive innovation approach, and found a balance between flexibility and formal structures of integration. For leadership and governance, they applied collaborative governance by engaging all stakeholders and distributed leadership throughout all levels of the system. Key enabling mechanisms also included ICT developed specifically to support collaboration and communication, and the creation of feedback loops and a continuous monitoring system [29]. The overarching finding was that alignment work — connecting the different components and levels of the health and social care system — is a prerequisite for successful implementation. A comparative study of integrated care policy supports in England, Germany, and the Netherlands similarly found that power imbalances between health and social services sectors impede collaboration, that data infrastructure challenges persist across all three systems, and that legislation is an important enabling factor for supporting governance [37].

The Finnish case aligns with and extends these international experiences in important ways. Like the Netherlands, Finland has defined statutory service packages and pathways as a mechanism for structuring integrated care for identified client groups. Like the UK, Finland faces challenges in translating formalised pathway structures into changed professional practice. And like the broader European experience documented in SELFIE, the Finnish findings confirm that structural reform alone is insufficient: implementation requires active governance, collaborative development, and monitoring mechanisms that connect policy objectives with everyday service delivery. What distinguishes the Finnish case is the legally mandated scope of integration — encompassing both health and social care across all client groups requiring multidisciplinary support — and the novelty of the governance structures in which this is being implemented, which creates both opportunity and instability in the early implementation phase.

### Key insights from the case

The study suggests that predefined service packages and pathways can function as an important implementation tool for integrated care. By making service structures, client groups, and collaboration practices explicit, they support a shared understanding among professionals and managers and provide a framework for coordinating services across organisational boundaries. This finding aligns with international evidence highlighting the importance of care pathways and shared frameworks in supporting integrated care for people with complex needs [5,4].

At the same time, the findings underline that defining service packages and pathways is only an initial step. Their practical value depends on how they are embedded in governance structures, steering mechanisms, and everyday professional practices. In this study, steering and monitoring arrangements were often underdeveloped, reflecting both the early stage of implementation and the limited availability of data to support knowledge-based management. Similar challenges have been reported in other integrated care initiatives, where monitoring and evaluation often lag behind service design [29].

### Implementation challenges

The challenges identified in this study mirror those widely discussed in the integrated care literature. Fragmentation of services, strong professional boundaries, and difficulties in aligning organisational goals were common barriers to implementation [19,20]. These challenges were intensified by the reform context, where counties were required to develop new practices while simultaneously managing financial pressures and organisational change [15].

A particularly salient finding concerns the role of management and governance. The development of multidisciplinary service packages and pathways required active managerial involvement, clear allocation of responsibilities, and horizontal coordination across service sectors. Without such support, professionals were often left to navigate complex collaboration arrangements on their own, limiting the effectiveness of integration efforts. The importance of appointing process owners with sufficient decision-making authority emerged as a key enabling factor, echoing international calls for stronger leadership and accountability structures in integrated care [29].

### Implications for integrated care development

The findings highlight the value of using structured development models, such as the co-creation framework applied in this study, to guide integrated care implementation. By linking definition, steering, and monitoring into a continuous process, the model helps to clarify the different dimensions of integration and identify areas requiring further development. This approach supports the view of integrated care as an adaptive process that evolves over time through learning and feedback rather than a one-time organisational change.

However, the study also demonstrates that integrated care solutions cannot be fully standardised. While common elements of service packages and pathways can be identified across counties, their implementation is shaped by local organisational arrangements, professional cultures, and available resources. This reinforces the importance of balancing national-level guidance with flexibility for local adaptation, a challenge observed in many integrated care reforms internationally [31,29].

### Relevance beyond the Finnish context

Although situated in a specific national reform context, the challenges and enabling factors identified here are not unique to Finland. Many health and social care systems are seeking ways to improve coordination for clients with complex needs while navigating organisational fragmentation and professional boundaries. Rather than a model to be replicated, the Finnish case provides transferable insights into the processes, conditions, and governance arrangements that support integrated care in practice — highlighting the importance of implementation-focused approaches that connect policy objectives, organisational structures, and everyday professional work.

## Lessons learned

- Predefined multidisciplinary service packages and pathways can support integrated care, particularly for clients with complex and multi-sectoral needs. By clarifying service structures, roles, and collaboration practices in advance, they help professionals identify clients who benefit from integration and coordinate support more coherently.
- Service packages and pathways are implementation tools for integrated care, not only solutions in themselves. Their value depends on how they are embedded in everyday professional practice and supported by organisational governance, leadership, and shared goals across sectors.
- Managerial support and clear governance structures are essential. Integrated care development requires active leadership, horizontal coordination across service sectors, and the allocation of responsibility, for example through appointing a dedicated process owner with sufficient decision-making authority.
- Strong professional identities and siloed organisational cultures remain key barriers. Moving towards integrated care requires sustained efforts to support multidisciplinary collaboration, shared understanding, and trust between professionals and organisations, beyond formal guidelines or models.
- Knowledge-based steering and monitoring often lag behind service development. Limited availability of shared data and indicators constrains the ability to guide and evaluate integrated care, highlighting the need to strengthen monitoring and learning mechanisms alongside service design.
- National guidance benefits from local adaptability. While common elements of service packages and pathways can be identified and shared, their implementation must remain responsive to local contexts, resources, and professional cultures.
- Integrated care develops incrementally over time. Early phases are characterised by experimentation, negotiation, and incomplete structures; realistic expectations and long-term commitment are required for integration to become embedded in routine practice.

## Conclusions

This study examined the early development and implementation of multidisciplinary service packages and pathways in Finnish wellbeing services counties following a major system-level reform. The findings demonstrate that predefined service packages and pathways can function as practical implementation tools for integrated care, particularly for clients with complex and multi-sectoral needs. By clarifying service structures, roles, and modes of collaboration, they support professionals in planning and delivering more coherent and coordinated individual service pathways.

At the same time, the study highlights that service packages and pathways are not a standalone solution to fragmentation. Their effectiveness depends on how they are embedded in governance structures, managerial practices, and everyday professional work. In the early stages observed in this study, much of the development effort focused on defining service content and collaboration arrangements, while steering and monitoring mechanisms remained underdeveloped. This underlines the importance of treating definition, steering, and monitoring as interdependent dimensions of integrated care rather than sequential steps.

The findings emphasise the central role of leadership and governance in enabling integrated care. Active managerial involvement, horizontal coordination across sectors, and clearly assigned responsibility — such as a designated process owner with decision-making authority — are crucial for sustaining multidisciplinary collaboration. Without these conditions, service packages and pathways risk remaining abstract models with limited influence on practice.

Integrated care development is also shaped by professional cultures and organisational histories. Strong sectoral boundaries and established practices can hinder collaboration even in structurally integrated systems, requiring long-term commitment to cultural change, shared learning, and trust-building alongside formal tools and structures.

While situated in a specific national reform context, the lessons identified are relevant for other health and social care systems seeking to strengthen integrated care. The Finnish case illustrates how predefined service packages and pathways, developed through co-creation and supported by shared governance, can help translate integration policies into practice — contributing transferable insights into the conditions, processes, and challenges involved. Follow-up research will be needed to assess how service packages and pathways evolve over time, how steering and monitoring practices are established, and how clients and professionals experience integrated care in practice.
